# Ectomycorrhizal fungi alter soil food webs and the functional potential of bacterial communities

**DOI:** 10.1128/msystems.00369-24

**Published:** 2024-05-08

**Authors:** Louis Berrios, Glade D. Bogar, Laura M. Bogar, Andressa M. Venturini, Claire E. Willing, Anastacia Del Rio, T. Bertie Ansell, Kevin Zemaitis, Marija Velickovic, Dusan Velickovic, Peter T. Pellitier, Jay Yeam, Chelsea Hutchinson, Kent Bloodsworth, Mary S. Lipton, Kabir G. Peay

**Affiliations:** 1 Department of Biology, Stanford University, Stanford, California, USA; 2 Kellogg Biological Station, Michigan State University, Hickory Corners, Michigan, USA; 3 Department of Plant Biology, University of California, Davis, Davis, California, USA; 4 School of Environmental and Forest Sciences, University of Washington, Seattle, Washington, USA; 5 Division of CryoEM and Bioimaging, SSRL, SLAC National Accelerator Laboratory, Menlo Park, California, USA; 6 Earth and Biological Sciences Directorate, Pacific Northwest National Laboratory, Richland, Washington, USA; 7 Department of Earth System Science, Stanford University, Stanford, California, USA; State Key Laboratory of Mycology, Institute of Microbiology, Chinese Academy of Sciences, Beijing, China

**Keywords:** bacteria-mycorrhizal fungi interactions, tripartite interactions, forest ecology, multi-omics, soil food webs, bacterial taxa-function relationships

## Abstract

Most of Earth’s trees rely on critical soil nutrients that ectomycorrhizal fungi (EcMF) liberate and provide, and all of Earth’s land plants associate with bacteria that help them survive in nature. Yet, our understanding of how the presence of EcMF modifies soil bacterial communities, soil food webs, and root chemistry requires direct experimental evidence to comprehend the effects that EcMF may generate in the belowground plant microbiome. To this end, we grew *Pinus muricata* plants in soils that were either inoculated with EcMF and native forest bacterial communities or only native bacterial communities. We then profiled the soil bacterial communities, applied metabolomics and lipidomics, and linked omics data sets to understand how the presence of EcMF modifies belowground biogeochemistry, bacterial community structure, and their functional potential. We found that the presence of EcMF (i) enriches soil bacteria linked to enhanced plant growth in nature, (ii) alters the quantity and composition of lipid and non-lipid soil metabolites, and (iii) modifies plant root chemistry toward pathogen suppression, enzymatic conservation, and reactive oxygen species scavenging. Using this multi-omic approach, we therefore show that this widespread fungal symbiosis may be a common factor for structuring soil food webs.

**IMPORTANCE:**

Understanding how soil microbes interact with one another and their host plant will help us combat the negative effects that climate change has on terrestrial ecosystems. Unfortunately, we lack a clear understanding of how the presence of ectomycorrhizal fungi (EcMF)—one of the most dominant soil microbial groups on Earth—shapes belowground organic resources and the composition of bacterial communities. To address this knowledge gap, we profiled lipid and non-lipid metabolites in soils and plant roots, characterized soil bacterial communities, and compared soils amended either with or without EcMF. Our results show that the presence of EcMF changes soil organic resource availability, impacts the proliferation of different bacterial communities (in terms of both type and potential function), and primes plant root chemistry for pathogen suppression and energy conservation. Our findings therefore provide much-needed insight into how two of the most dominant soil microbial groups interact with one another and with their host plant.

## INTRODUCTION

The interactions between plants and ectomycorrhizal fungi (EcMF) have begun to emerge as critical factors for generating differences in the structure of terrestrial ecosystems. EcMF and land plants form one of the most widespread and ecologically relevant mutualisms on Earth, exchanging vital organic resources and improving one another’s growth under most natural conditions (1–5). On the ecosystem scale, there is now clear evidence that the presence of EcMF symbioses significantly alters local nutrient economies (6–8). These changes can have important consequences, for example, that result in increased soil carbon sequestration, an increased C:N ratio in plant tissues (9–12), and an increased capacity for plants to respond to eCO_2_ (13, 14). Evidence is also starting to emerge that EcMF alter other important aspects of ecosystem structure, such as biological interactions (15–17). For example, bacterial community richness and composition appear to vary with the presence of EcMF (18). It is unclear though whether these changes are simply due to changes in the soil environment (e.g., pH and C:N ratio) associated with EcM forests (6) or are the result of direct interactions with EcMF. If EcMF have generalizable effects on biological interactions in soils, then this would have wide-ranging implications for our understanding of the soil microbiome.

The growth dynamics and lifestyle strategies of EcMF suggest that EcMF can alter microbial interactions in and around root systems (19). In general, microbial composition and function are known to differ in soils based on their proximity to roots (20, 21). The area near the root surface (often called the “rhizosphere”) differs in the quality and quantity of resources, and microbes that are further away from plant roots have reduced access to these resources (22). The nature of EcM symbiosis suggests that it should have distinct effects on soil microbial communities compared to the standard rhizosphere view of non-ectomycorrhizal plants. First, EcM symbioses develop a thick fungal mantle that completely envelops apical plant roots, where root exudate production and nutrient uptake are normally greatest. Second, the foraging mycelia (or extraradical mycelium) are highly developed and can extend long distances into the surrounding soil, disrupting clear divisions between soil and root-associated microbial communities (18, 23). The collection of these well-established facts thus provides strong evidence for the idea that “mycorrhizal highways” may produce and distribute metabolites that shape plant and soil microbial community dynamics (24, 25). Yet, relatively few studies have investigated the effect(s) that the presence of EcMF has on soil resources and community structure (26). Consequently, much of what we know about the microbial relationships that develop in this expanded soil-root region—known as the hyphosphere—remains associational (23).

Though limited, multiple lines of evidence support the notion that EcMF can transport plant-derived resources and restructure bacterial communities. For example, tree girdling experiments—a technique that terminates rhizodeposition—performed in natural forest environments have shown that mycorrhizal fungi may transport more than 60% of rhizodeposits to the soil (27). A meta-analysis of 26 field studies across 43 sites showed that the hyphae of EcMF increase the potential for resource distribution by roughly four orders of magnitude in the soil relative to non-ectomycorrhizal plant roots (23). Likewise, greenhouse experiments have supported these field observations. For instance, Zhou et al. (28) found that ectomycorrhizas transport ~7% more carbon-labeled rhizodeposits to soil than non-ectomycorrhizal roots. Other greenhouse studies have similarly shown that ectomycorrhizas facilitate rhizodeposit distributions (29, 30). The proportion of studies that investigate how ectomycorrhiza-mediated rhizodeposition alters bacterial community composition is comparably low (31), yet strong evidence suggests that arbuscular mycorrhizal fungi (AMF) may drive the structure and function of soil bacterial communities (32–35). Considering the similarities between arbuscular and ectomycorrhizal symbioses (1), it is likely that the presence of EcMF can restructure soil bacterial communities and the composition of belowground metabolites. It has nonetheless remained a challenge for the field to align EcMF-associated changes to belowground metabolites with shifts in bacterial community structure.

In a recent field survey, we found that EcMF form consistent associations with soil bacteria across much of the geographic and climatic ranges of the host tree *Pinus muricata* (18). In the same study, we used microcosms to show that these associations appear to have tangible effects on plant and fungal fitness (18) and that *Suillus pungens—*a common EcMF in this system—recapitulates many of the dominant bacterial associations observed in the field. While evidence is becoming clear that the presence of EcMF should have strong effects on bacterial community structure, little is still known about the mechanisms that mediate interactions between EcMF and soil bacteria. Some reports have implicated antibiotics (36), chemical changes to the soil environment (23), and changes to microbial signaling pathways, metabolism, cell structure, and cell growth responses as drivers of these interactions (37). Others have shown that biofilm formation and organic acids also play a role (38, 39). Yet, controlled inoculations with and without EcMF and multi-omics approaches are required to determine how the presence of EcMF can alter belowground metabolites and bacterial community structure.

To address these uncertainties, we used a simplified growth chamber environment to experimentally test whether the presence of EcMF changes bacterial community structure and belowground metabolite composition. To this end, we grew *P. muricata* plants in either the presence or absence of a common EcMF (*S. pungens*), characterized bacterial communities, and profiled belowground metabolites. Our analyses demonstrate that the presence of EcMF increases bacterial richness, selects for a more homogenous pool of bacteria, shapes the metabolic potential of bacterial communities, and fosters the production of unique metabolites that appear to function as both resources and mediators of soil interactions. We have therefore begun to reveal how EcMF restructure soil organic resources and bacterial communities, which suggests that EcMF modify soil communities and resources wherever EcM plant communities occur.

## MATERIALS AND METHODS

### Plant growth experiments and microbial inoculations

To prepare for our plant growth experiments, *Pinus muricata* D.Don (Bishop pine) seeds were first collected in Point Reyes National Seashore (PRNS). They were surface sanitized with a 4.5% H_2_O_2_ and Nanopure water solution and 100 µL of Tween-80 for 20 minutes. Then, they were rinsed and soaked with Nanopure water for 24 hours to remove excess debris and the remaining chemicals. Afterward, the seeds were placed on moistened filter paper for 3 weeks in the dark to germinate. Seedlings were then transplanted and grown in autoclaved 99% quartz sand (Thermo Scientific Chemicals, 40–100 mesh)—housed in individual Cone-tainers (Ray-Leach)—instead of natural soils to minimize the effects of legacy DNA or microbial by-products that could obscure treatment effects. Two months after germination, the seedlings were treated with a bacterial wash. The bacterial wash was prepared by collecting fresh soil from Bishop pine forests in PRNS (stored at 4°C until use), suspending 5 g in 45 mL of Nanopure water, vortexing the soil-water mixture for 1 minute, centrifuging the mixture at 600 × *g* for 2 minutes, and then filtering the supernatant using a 1 µm Luerlock syringe filter (Whatman Puradisc PTFE). A total of 10 mL of the filtrate was added to each Cone-tainer using a syringe. The seedlings were treated with an initial pulse of 30 mL of Ingestad solution (40), saturated with deionized water (diH_2_O) once weekly, and grown in a growth chamber at 23°C under a 12:12 hour light:dark cycle with 60% humidity.

Ectomycorrhizal spores and spore-free diH_2_O solutions were prepared for plant inoculations by first collecting spores from fresh sporocarps collected from PRNS. Mushroom caps (pores down) were placed on tinfoil to facilitate spore dispersal on the tinfoil. After 36 hours, the spores were suspended in diH_2_O. The spore solution was diluted to a known spore concentration using a hemocytometer, and the seedlings were inoculated with either *Suillus pungens* spores (5 × 10^6^ spores per seedling) or equal volumes of spore-free diH_2_O solution 7 weeks post-germination.

To allow for well-developed mycorrhizas, seedlings were harvested 5 months after they were inoculated with spores or the spore-free solution. We uprooted each seedling, gently shook off the roots, and homogenized the soil in an ethanol-sanitized bin. Subsamples were then flash-frozen in liquid nitrogen for DNA and RNA extraction (2.0 ± 0.1 g) and omics analyses. Because algae had grown on the surfaces of the sand medium in many pots, the top ~5 mm of each soil sample was discarded. After collecting soil samples, root systems were rinsed of adhering soil and examined under a dissecting microscope (~40×) to confirm the colonization status of the roots. For spatially resolved metabolite and lipid analysis, we harvested the terminal ~1 cm of fine root clusters and froze them in liquid nitrogen. For plants colonized by mycorrhizal fungi, we selected roots with clusters of swollen mycorrhizas that had an evident fungal mantle, no root hairs, and emanating hyphae. For uncolonized plants, we selected root samples with firm, light-colored root tips that had many root hairs. Five such clusters per plant and condition were collected and frozen for downstream analyses.

### Nucleic acid extractions and 16S rRNA amplicon library preparation

A mass of 2 g of sand-soil material was subsampled from each microcosm’s total soil volume, frozen in liquid nitrogen, and stored individually in a 15 mL Falcon tube at −80°C until DNA and RNA extractions were performed. Total nucleic acids were extracted using an RNeasy PowerSoil Total RNA Kit (Qiagen, Carlsbad, CA, USA). We modified the extraction protocol because the low amount of microbial biomass in our microcosms prevented us from extracting sufficient nucleic acids for downstream analyses of both DNA and RNA from all samples. Therefore, we chose to eliminate the RNA filtering step, which allowed us to remove cellular and soil debris while preserving all captured nucleic acids (DNA and RNA). To account for the potential differences between microbial abundance and activity, we used half of our samples for downstream DNA sequencing and half for downstream RNA sequencing. DNA was removed from samples allocated for RNA sequencing using a DNase Max Kit (Qiagen, Carlsbad, CA, USA), and RNA was removed from samples for DNA sequencing using RNase A (Qiagen, Carlsbad, CA, USA). All samples were analyzed with a Qubit Fluorometer 3.0 (Thermo Fisher, Waltham, MA, USA) to quantify the focal nucleic acid and ensure that all remnant DNA or RNA was removed accordingly. DNA samples were stored at −20°C until they were needed for library preparation. RNA samples were converted to cDNA using a High-Capacity cDNA Reverse Transcription Kit (Applied Biosciences) according to the manufacturer’s protocol. Resultant cDNA constructs were quantified using a Qubit Fluorometer 3.0 (Thermo Fisher, Waltham, MA, USA) to ensure only DNA was present. The samples were then stored at −20°C until they were needed for library preparation.

PCR of the resulting DNA and cDNA was performed to identify bacterial communities. First-step PCR amplifications were performed by mixing 1 µL of template DNA, 5 µL of MyTaq HS Red Mix, 3.2 µL of PCR-grade H_2_O, and 0.4 µL of each primer (515f/806R primer pair to amplify the V4 hypervariable region of the bacterial 16S rRNA gene) (41). PCR amplifications were carried out in 96-well plates and consisted of 35 cycles with a denaturation temperature of 95°C, an annealing temperature of 50°C, and an extension temperature of 72°C. Indexed tags for Illumina sequencing were attached during a second step PCR amplification (42), which consisted of eight cycles with identical temperature settings as the first step PCR. Gel electrophoresis was used to confirm PCR products, and a magnetic bead purification method using Sera-Mag SpeedBeads (MilliporeSigma, Munich, Germany) was used to clean the resulting PCR products. A Qubit Fluorometer 3.0 (Thermo Fisher, Waltham, MA, USA) was used to quantify cleaned DNA. Finally, we pooled our samples into two libraries at equimolar concentrations and submitted them for 2 × 300 Illumina MiSeq sequencing at the Stanford Functional Genomics Facility. Raw sequences are available from NCBI Short Read Archive under the BioProject accession number PRJNA1100605.

### Amplicon sequencing bioinformatics

We received 1,895,332 demultiplexed reads (average per sample = 52,648). After filtering, denoising, merging forward and reverse reads, and removing chimeric sequences using the DADA2 workflow (43), our 16S rRNA data set consisted of 934,306 reads (average per sample = 26,694). The DADA2 workflow accuracy was evaluated by inferring and matching mock community members to their expected sequences (residual error rate = 0%). Taxonomic classifications were assigned to bacteria using the SILVA database version 138.1 with the naive Bayesian classifier (44). Sequences assigned to “chloroplast” and “mitochondria” were removed from our data set, and we removed taxa with less than 10 sequences and removed samples with less than 1,000 sequences. Samples were rarefied to a read depth of 5,000 using the rarefy_even_depth function in the phyloseq package in R (45). We retained 28 samples in our data set (DNA = 12; RNA = 16), which consisted of 572 amplicon sequence variants (ASVs). We then used PICRUSt2 (46), which matches 16S-based taxonomy to the nearest sequenced reference genomes, to estimate the functional profiles for our identified ASVs. Both KEGG ortholog (KO) and enzyme commission numbers (EC) pathway-level matching were performed to report maximal functional estimate information from our PICRUSt2 analysis. Since PICRUSt2 extrapolates information from reference genomes, outputs should be viewed as rough proxies for maximum functional potential rather than a direct measure of *in situ* activity. PICRUSt2 outputs were used in tandem with MIMOSA2 (47) to estimate community metabolic potentials (CMPs) of the soil bacterial communities. MIMOSA2 combines reference genome information and microbiome compositional data from PICRUSt2 with metabolite information and a reaction database to generate community metabolic models that assess whether measured metabolite concentrations are consistent with estimated CMP across a set of samples. Using this information, MIMOSA2 then identifies specific taxa and reactions that can explain observed metabolite variation. Bergey’s manual was used to assign cell motility and cell wall structure information to bacterial taxa (48).

### Statistical analyses of amplicon sequence data

Statistical analyses were performed in R studio version 4.2.1 (49). The phyloseq package was used to derive alpha and beta diversity estimates (45). The microbiome package was used to transform our abundance data and derive relative abundance measures (50). We tested for differences in bacterial community composition between experimental conditions (i.e., with *S. pungens* [EcMF]) or without *S. pungens* [No EcMF]) and transcriptionally active (RNA) versus total (DNA) bacterial communities using a permutational analysis of variance (PERMANOVA) as implemented in the adonis2 function from the vegan package (51). The betadisper function was used to test for homogeneity of variances across treatment groups. The DESeq2 package was used to determine differential abundance(s) of microbial genera between experimental conditions (52). All plots were generated using either ggplot2 (53) or ggpicrust2 (54). All the code generated for these analyses can be obtained from https://github.com/LouisBerrios.

### Gas chromatography-mass spectroscopy metabolomics analysis

Soil samples were collected from around the roots of *Pinus muricata* (Bishop pine) plants, weighed, flash-frozen, and stored at −80°C prior to gas chromatography-mass spectroscopy (GC-MS) analysis. Dried metabolite extracts were derivatized with a modified version of the protocol used to create FiehnLib (55). First, the samples underwent methoximation to protect carbonyl groups and reduce tautomeric isomers. Then, silylation with N-methyl-N-trimethylsilyltrifluoroacetamide and 1% trimethylchlorosilane was performed to derivatize hydroxy and amine groups to trimethylsilated forms. Samples were analyzed in an Agilent GC 7890A coupled with a single quadrupole MSD 5975C (Agilent Technologies) over a mass range of 50–550 *m*/*z*. A standard mixture of fatty acid methyl esters (C8–C28) was analyzed in tandem for RI alignments. Samples were held at 60°C for 1 minute after injection, followed by 10°C temperature increases per minute till a maximum temperature of 325°C was reached and maintained for 5 minutes.

GC-MS raw data files were processed using Metabolite Detector (56). Agilent .D files were converted to netCDF format using Agilent Chemistation and then converted to binary files using Metabolite Detector. Retention indices of the detected metabolites were calculated based on the analysis of fatty acid methyl ester standard mixtures and chromatographic deconvolution and alignment. Initially, metabolites were identified by matching experimental spectra to an augmented version of FiehnLib (57). These initial identifications were then followed by manual validation using the NIST 14 GSMS library. The summed abundances of the three most abundant fragment ions of each identified metabolite were integrated across the GC elution profile (automatically determined by Metabolite Detector). Fragment ions due to trimethylsilylation (that is, *m*/*z* 73 and 147) were excluded from the determination of metabolite abundance. Features resulting from GC column bleeding were removed from the data set before further data processing and analysis. Processed GC-MS data were analyzed further in Matlab 2020b and pmartR (58). All samples with *m*/*z* scores of zero were replaced with NaN and weight-normalized values were log_2_ transformed for statistical analyses. Only metabolites that were present in three or more replicates were analyzed. To determine the community-wide differences in metabolites, we conducted NMDS ordinations and PERMANOVAs using the phyloseq package (45). We then used DESeq2 (52) to identify metabolites that were significantly enriched between conditions (EcMF versus No EcMF).

### MALDI-FTICR MS imaging analysis of ectomycorrhizas

Ectomycorrhizal and non-ectomycorrhizal roots were analyzed and compared using matrix-assisted laser desorption/ionization Fourier-transform ion cyclotron resonance mass spectrometry imaging (MALDI-FTICR MSI). Roots were cleaved from living plants and were immediately flash-frozen for downstream analyses. Three roots per treatment (ectomycorrhizal root or non-ectomycorrhizal root) were analyzed with two technical replicates. Roots were embedded within a mixture of 7.5% hydroxypropyl methylcellulose and 2.5% polyvinylpyrrolidone and cryosectioned using CryoStar NX-70 Cryostat (Thermo Scientific, Runcorn, UK). Longitudinal root sections (12 µm) were coated with N-(1-naphthyl) ethylenediamine dihydrochloride MALDI matrix using M5 Sprayer (HTX Technologies, Chapel Hill, NC, USA) to track small molecules in the tricarboxylic acid cycle, amino acids, carbohydrates and their conjugates, and organic and phenolic acids. All imaging analyses were performed in negative ionization mode with broadband excitation from *m*/*z* 92 to 1,000 and a resolving power of ~70,000 at 400 *m*/*z* on a Bruker Daltonics 15T solariX FTICR MS, equipped with a ParaCell, and MALDI source with a SmartBeam II frequency-tripled (355 nm) Nd: YAG laser (Bremen, Germany). A 35 µm step size was used, and data sets were acquired using FlexImaging (Bruker Daltonics). FlexImaging sequences were directly imported and statistically treated into the SCiLS lab (receiver operating characteristic [ROC] and colocalization analysis) and then submitted to METASPACE for molecular annotations using the KEGG database. ROC is a univariate measure to assess the discrimination quality of a feature for populations defined through two groups. The ROC was calculated based on the statistical specificity and sensitivity when the intensity of a single feature is used for the discrimination rule. The area under the ROC curve measures the discrimination quality in the interval between 0.0 and 1.0, where perfect discrimination would yield an area under the curve (AUC) equal to 1 or 0. As the first group, we selected all sections with ectomycorrhizae, and as the second group, we selected all sections without ectomycorrhizae. If the feature (*m*/*z*, metabolite) had an AUC value < 0.4 or >0.6, we considered it a discriminant.

## RESULTS

### Ectomycorrhizal fungi promote bacterial richness, homogenize bacterial communities, and alter the metabolic potentials of soil bacteria

**Fig 1:**
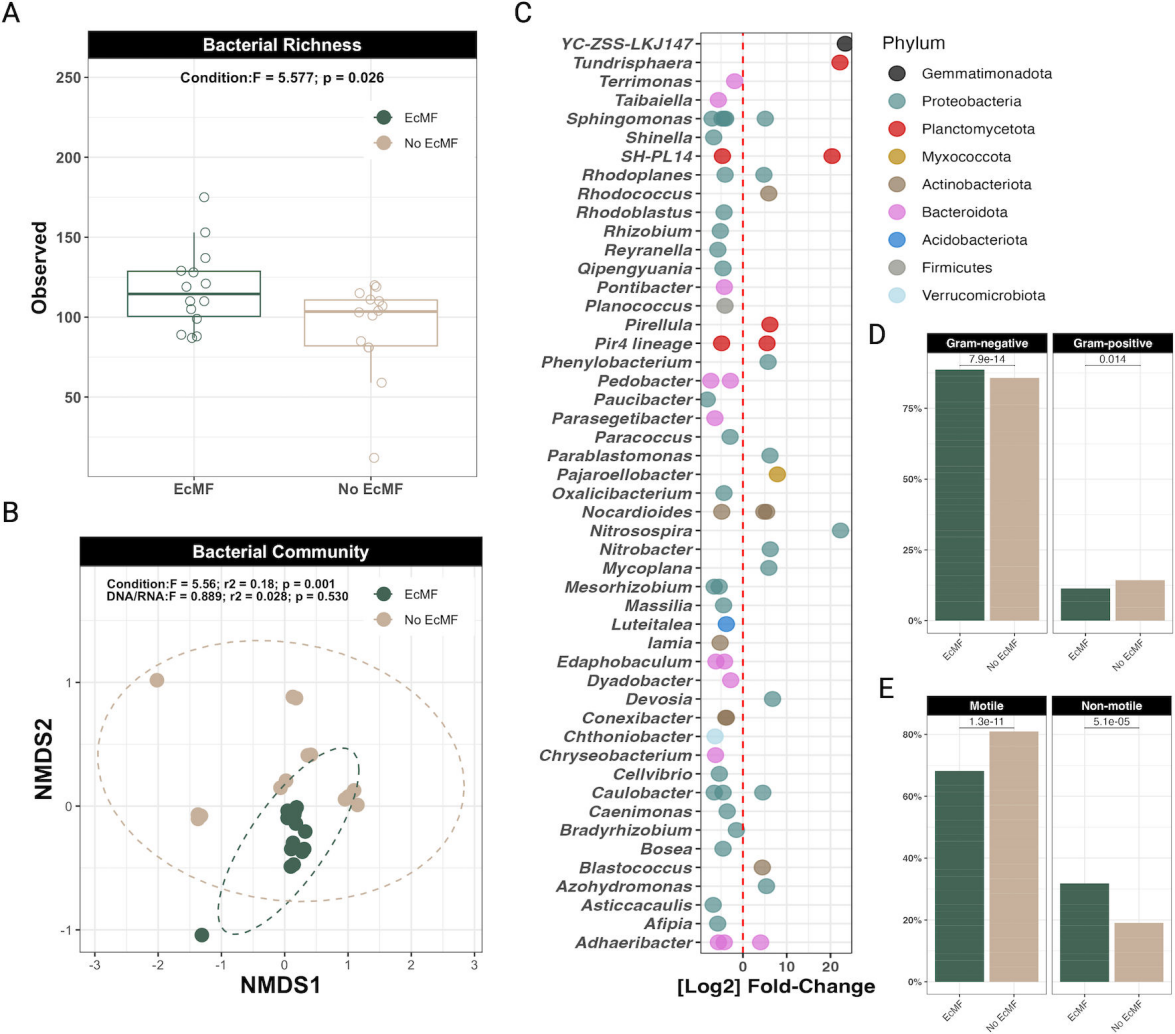
Diversity estimates of bacterial communities either with EcMF or without EcMF. (**A**) Observed bacterial richness between treatments (i.e., either with EcMF or without EcMF [No EcMF]). (**B**) Beta diversity comparison between conditions and library preparation type (i.e., from DNA templates or from RNA to cDNA). Stress = 0.0914. In both panels **A** and **B**, *n* = 13 for each condition. (**C**) DESeq2 analysis showing which ASVs are differentially abundant in either the presence of EcMF (left of the red dotted line) or the absence of EcMF (right of the red dotted line). Each ASV is denoted by a single dot. ASVs are grouped by genus on the *y*-axis and colored by phylum designation. All the ASVs shown are statistically enriched (*P* < 0.001), and all *P*-values were corrected using the Benjamini and Hochberg method (52). (**D**) Ratios of Gram-negative and Gram-positive bacteria that were differentially abundant according to DESeq2. (**E**) Ratios of motile versus non-motile bacteria that were differentially abundant according to DESeq2. *P* values are shown above each comparison (*t*-test, where *P* < 0.05 equals a significant difference between groups).

To determine how ectomycorrhizal fungi change bacterial community composition, we first compared alpha and beta diversity metrics between experimental treatments (i.e., plants grown with or without the EcMF *S. pungens*). We found that the presence of EcMF increased bacterial richness in soils compared to those without EcMF (*F*_1,26_ = 5.577; *P* = 0.026; Fig. 1A). When we tested the differences in beta diversity between the EcMF and non-mycorrhizal (NM) treatments using a PERMANOVA, we found that the bacterial communities differed significantly between these treatments (*F*_1,26_=5.56; *r*^2^ = 0.18; *P* = 0.001; Fig. 1B). The bacterial communities in the EcMF treatment were more similar to one another compared to bacterial communities without EcMF present, suggesting that the presence of EcMF selects for a taxonomically conserved group of bacterial taxa (Fig. 1B). A beta dispersion test further confirmed our observation that EcMF foster the assembly of more homogenous bacterial communities compared to those without EcMF (*F*_1,26_=15.04; *P* = 0.000642; Fig. S1). Moreover, we observed differences in both the cell wall structure (i.e., Gram-positive or Gram-negative) and motility potential between bacterial communities in the presence or absence of EcMF (Fig. 1D and E). There were no significant differences detected between microbial communities sequenced using DNA versus RNA (PERMANOVA: *F*_1,26_=0.889; *r*^2^ = 0.028; *P* = 0.530; beta dispersion: *F*_1,26_=0.0412; *P* = 0.841), suggesting that the bacteria detected from DNA were also metabolically active members of the community.

Next, we used DESeq2 (52) to determine which bacterial taxa were differentially enriched between experimental treatments. A total of 44 amplicon sequence variants were significantly enriched in soils with EcMF, whereas 21 ASVs were enriched in soils without EcMF. Of the 44 ASVs that were enriched in the EcMF treatment, 77% (34/44 total ASVs, spanning 60% of the differentially enriched genera detected) were enriched exclusively in EcMF-inoculated soils. The remaining 10 ASVs belonged to genera that were also enriched in soils without EcMF (Fig. 1C). Many of the EcMF-enriched ASVs matched to either the phylum Proteobacteria or Bacteroidota, whereas soils without EcMF were enriched with ASVs that mostly matched to either Proteobacteria or Planctomycetota (Fig. 1C; Fig. S2). Taken together, though we observed phyla- and genus-level taxonomic overlap between conditions, our data demonstrate that the presence of EcMF selects for distinct ASVs.

**Fig 2:**
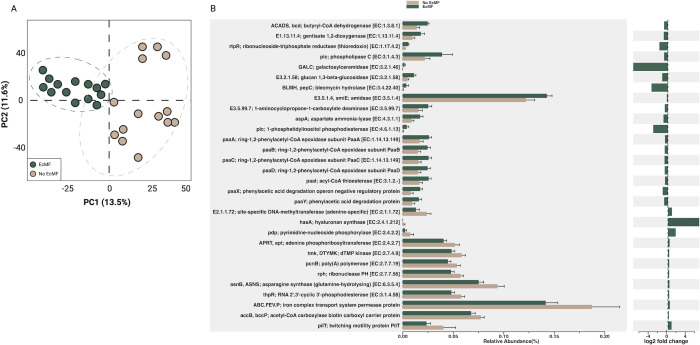
PICRUSt2 analysis of soil bacterial communities in the presence or absence of EcMF. (**A**) Principal component analysis demonstrating the functional differences between bacterial communities based on treatment. Ellipses indicate confidence intervals of 95%. (**B**) The top 30 KEGG features that were differentially abundant between treatments according to DESeq2 (*P* < 0.05; *P*-adjusted using the Holm-Bonferroni method). Error bars indicate confidence intervals of 95%. The relative log_2_ fold changes are provided adjacent to the relative abundance of the top 30 gene products, and EC numbers are listed alongside their corresponding gene product name. See Tables S1 and S2 for a comprehensive list of the KO features and EC numbers detected in our analyses.

To estimate the functional differences between bacterial communities with and without EcMF, we used PICRUSt2 (46). In line with our NMDS ordination (Fig. 1B), our principal component analysis showed clear functional differences between these bacterial communities at both the KO-pathway level (Fig. 2A; PC1 = 12.5%, PC2 = 11.9%) and EC-number level (Fig. S2A; PC1 = 13.5%, PC2 = 11.6%). Likewise, compared to the bacterial communities without EcMF, the degree of bacterial potential functions was more similar in the EcMF-amended soils compared to soils without EcMF (Fig. 2A; Fig. S3A). Next, we sought to clarify these observed trends by determining which genes were differentially abundant between our experimental treatments. To do this, we mapped EC and KO numbers to biological functions and applied DESeq2 (52) to identify genes with the greatest variance between treatments (*P*-values adjusted using the Holm-Bonferroni method). A total of 376 unique KO features (Table S1) and 208 unique EC numbers (Table S2) were differentially abundant (*P* adjusted <0.05) between conditions. Key bacterial genes involved in plant-microbe symbioses were enriched in our EcMF treatment relative to our No EcMF treatment (based on inferred genome content from PICRUSt2). For example, the PICRUSt2 analysis suggests that the gene encoding 1-aminocyclopropane-1-carboxylate deaminase—which regulates plant ethylene levels—was enriched in our EcMF treatment (Fig. 2B). Likewise, the operon involved in the degradation of phenylacetic acid (*paa*A, *paa*B, *paa*C, *paa*I, *paa*X, and *paa*Y) was also likely enriched in our EcMF condition. Moreover, bacterial communities in the presence of EcMF were also inferred to harbor more genes to biosynthesize the carbohydrate trehalose (Fig. S3B). Together, our data highlight the potential for significant functional variations between the bacterial communities with EcMF present and those without EcMF present.

### The presence of ectomycorrhizal fungi alters soil food webs

**Fig 3:**
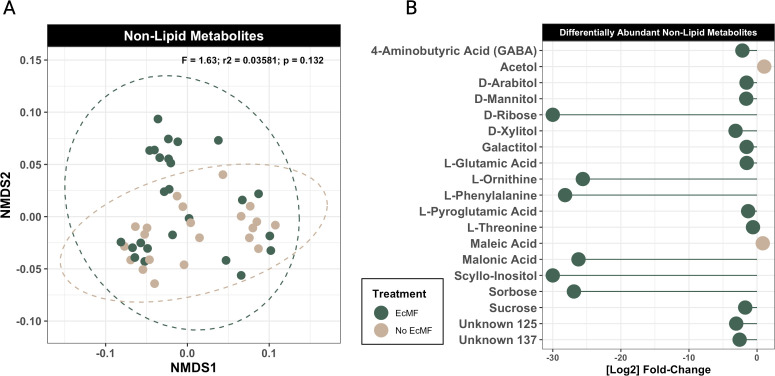
Soil metabolite comparison between soils with and without EcMF. (**A**) NMDS ordination plot comparing soil metabolites with (green) and without (tan) the addition of EcMF. PERMANOVA results are indicated in the upper right corner of the figure panel. (**B**) DESeq2 analysis illustrating the metabolites that are enriched in the presence of EcMF (left of the zero on the *x*-axis) and the absence of EcMF (right of the zero on the *x*-axis). All metabolites shown have a *P*-adjusted value (Benjamini-Hochberg) equal to or less than 0.05.

**Fig 4:**
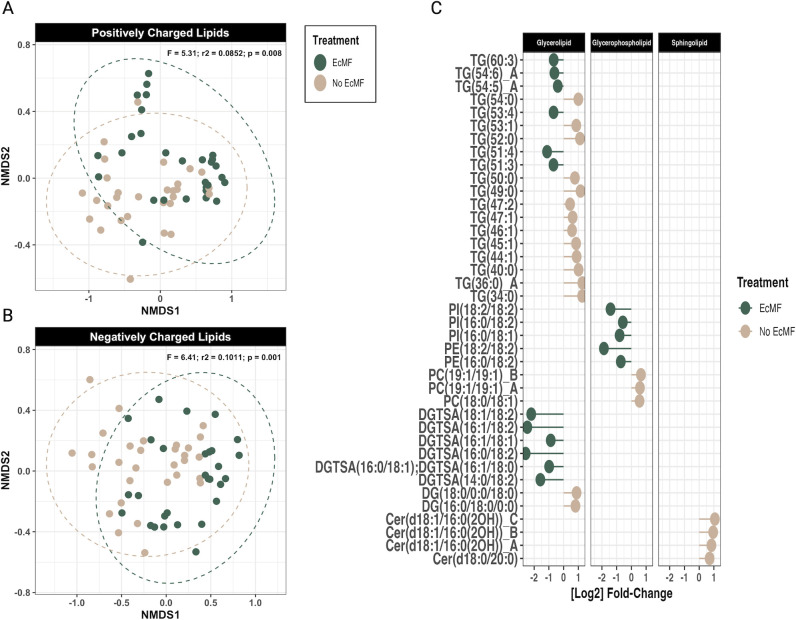
Soil lipid comparison between soils with and without EcMF. (**A**) NMDS ordination plot of the positively charged lipids and (**B**) negatively charged lipids. Soils with EcMF are colored green, and soils without EcMF are colored tan. PERMANOVA results are shown in the upper right corner of the NMDS figure panels. (**C**) DESeq2 analysis of the lipids that are enriched in the presence of EcMF (left of the zero on the *x*-axis) and in the absence of EcMF (right of the zero on the *x*-axis). All lipids shown have a *P*-adjusted value (Benjamini-Hochberg) equal to or less than 0.05. The lipid species designations are as follows: Cer, ceramide; HexCer, hexosylceramide; PC, phosphatidylcholine; PC-Lyso, lyso-phosphatidylcholine; PE, phosphatidylethanolamine; PG, phosphatidylglycerol; PI, phosphatidylinositol; DGDG, digalactosyldiacylglycerol; DGTSA, diacylglyceryltrimethylhomo-serine; SQDG, sulfoquinovosyl diacylglycerol; DG, diacylglycerol; and TG, triacylglycerol.

To understand how the presence of EcMF and their associated bacteria modify the biochemical composition of resources in soil food webs, we compared the metabolic and lipidomic profiles of soils with and without EcMF. To this end, we first performed NMDS ordinations on our metabolomic and lipidomic data sets. The overall metabolomic profiles of the soils with and without EcMF did not differ significantly (Fig. 3A; PERMANOVA: *F*_1,44_ = 1.63, *P* = 0.132). In contrast, the lipidomic profiles between these two conditions were significantly different for both positively (PERMANOVA: *F*_1,57_ = 5.31; *P* = 0.007) and negatively (PERMANOVA: *F*_1,57_ = 6.41; *P* = 0.001) charged lipids (Fig. 4A and B).

Next, we used DESeq2 (52) to identify individual soil metabolites that were differentially abundant between conditions with and without EcMF present. Of the 200 metabolites detected, only 19 metabolites were differentially abundant between our experimental conditions. Of these 19 metabolites, 17 were enriched in the presence of EcMF. The remaining two metabolites were enriched in the absence of EcMF. Six metabolites—D-ribose, L-ornithine, L-phenylalanine, malonic acid, scyllo-inositol, and sorbose—were highly enriched in the presence of EcMF (greater than a log_2_ fold change of 20), and the remaining 11 EcMF-enriched metabolites were moderately enriched (Fig. 3B). In total, one dicarboxylic acid (malonic acid), two unknown metabolites, three sugars (D-ribose, sorbose, and sucrose), five sugar alcohols (D-arabitol, D-mannitol, D-xylitol, galactitol, and scyllo-inositol), and six amino acids (L-glutamic acid, L-ornithine, L-phenylalanine, L-pyroglutamic acid, L-threonine, and 4-aminobutyric acid) were enriched in the presence of EcMF. Only one sugar alcohol (acetol) and one dicarboxylic acid (maleic acid) were enriched in the absence of EcMF. In addition, D-sorbose, D-ribose, and scyllo-inositol were consistently and exclusively detected in soils with EcMF (Fig. S4). Importantly, each of these three metabolites can be used as a primary carbon source to drive energy-intensive metabolic functions. Together, our soil metabolite data suggest that a large degree of metabolite overlap exists in soils with and without EcMF, yet the presence of EcMF is associated with the production of some unique soil metabolites that can be important bacterial resources.

We then used the same DESeq2 approach to determine which lipids were differentially abundant between soils with and without EcMF. A total of 216 lipids were detected in our initial profiling analysis. More than 80% (*n* = 174) of them were positively charged lipids such as ceramides and some sphingolipids, whereas the other ~20% were negatively charged lipids (*n* = 42). Of these 216 lipids, 17 lipids were significantly enriched in the presence of EcMF, and 22 lipids were significantly enriched in the absence of EcMF (Fig. 4C; adjusted *P* < 0.05). All the EcMF-enriched lipids were either glycerophospholipids or glycerolipids. Most of the glycerophospholipids comprised linoleic, oleic, and palmitic lipid tails typical of membrane-derived diacyl phospholipids (*n* = 11). The soil lipid profiles in the absence of EcMF were comparatively more diverse, including many unique sphingolipids, glycerophospholipids, and glycerolipids. Those glycolipids enriched tended to be monoacyled with tail lengths of 40–54 carbon atoms. To sum up, these lipidomic data suggest that soils with and without EcMF share many of the same lipids with none of the lipids we detected present exclusively in either of the two environments (Fig. S5). However, the presence of EcMF enriches select lipids (greater than a log_2_ fold change of 2) compared to those observed in soils without EcMF (Fig. 4C). Therefore, the enrichment of these lipids—which support metabolic and structural functions for plant and microbial life—may facilitate the divergent bacterial taxa-function relationships that we observed (Fig. 2).

### Bacterial taxa that are linked to soil resources

To clarify the link(s) between soil bacteria and soil resources, we estimated soil bacterial community metabolic potentials using MIMOSA2 (47) and compared CMPs between conditions. A total of 16 metabolites were linked to bacterial metabolic processes (Table S3), and six of these (i.e., dihydroxyacetone, fumaric acid, phthalic acid, succinic acid, L-threonine, and L-valine) had CMPs that were significantly different (Welch’s *t*-test; *P* < 0.05) between conditions (Fig. S6). One additional metabolite, caprylic acid, was linked exclusively to bacterial communities from soils amended with EcMF. The remaining nine metabolites—L-glutamic acid, glycine, L-lactic acid, palmitic acid, glyceric acid, maleic acid, stearic acid, pyroglutamic acid, and adipic acid—did not have CMPs that differed significantly between conditions (*P* > 0.05), suggesting that these metabolites may be common resources that are shared among bacterial taxa and that they are not largely determined by the presence of EcMF.

**Fig 5:**
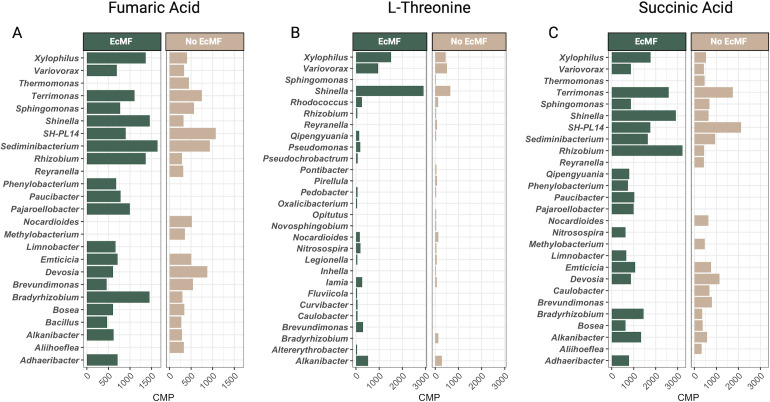
Strongly associated community metabolic potentials derived from MIMOSA2 metabolite-taxa predictions. CMPs for individual amplicon sequence variants were aggregated at the genus level, and the CMPs for the top 20 bacterial genera are shown for (**A**) fumaric acid, (**B**) L-threonine, and (**C**) succinic acid.

Next, we matched ASVs to their CMPs and aggregated CMPs at the genus level to determine which bacterial genera were significantly associated with soil chemistry. Since the MIMOSA2 CMP outputs can be either negative (low correlation between input metabolite data and taxonomic data) or positive values (high correlation between input metabolite data and taxonomic data), we focused on the positive CMP values (i.e., those that likely play strong roles in modifying soil food webs). However, a comprehensive list of all metabolites, their CMPs, and their matched taxa can be found in Table S3. Three metabolites—fumaric acid, L-threonine, and succinic acid—were represented significantly by the CMP predictions of MIMOSA2, and several taxa were consistently among the top 20 bacterial genera that were positively associated with these metabolites (Fig. 5). For instance, *Bradyrhizobium*, *Rhizobium*, and *Shinella* were significantly associated with these metabolites in the presence of EcMF. Members in the genera *Caulobacter*, *Hydrocarboniphaga*, and *Qipengyuania* were the only taxa with predicted contributions to caprylic acid, and they were present exclusively in the presence of EcMF. In contrast, the metabolite phthalic acid was associated with seven bacterial genera (*Rhizobium*, *Psychroglaciecola*, *Iamia*, *Hyphomicrobium*, *Fimbriimonas*, *Devosia*, and *Bacillus*). Of these, only *Rhizobium*, *Hyphomicrobium*, *Devosia*, and *Bacillus* had positive CMPs, and their CMPs were specific to our EcMF condition. The CMPs for *Psychroglaciecola* and *Fimbriimonas* were negative and observed in both conditions (i.e., EcMF and No EcMF), and the CMP for the genus *Iamia* was positive but only detected in the absence of EcMF.

### Ectomycorrhizal fungi modify the chemical environments in and around roots

**Fig 6:**
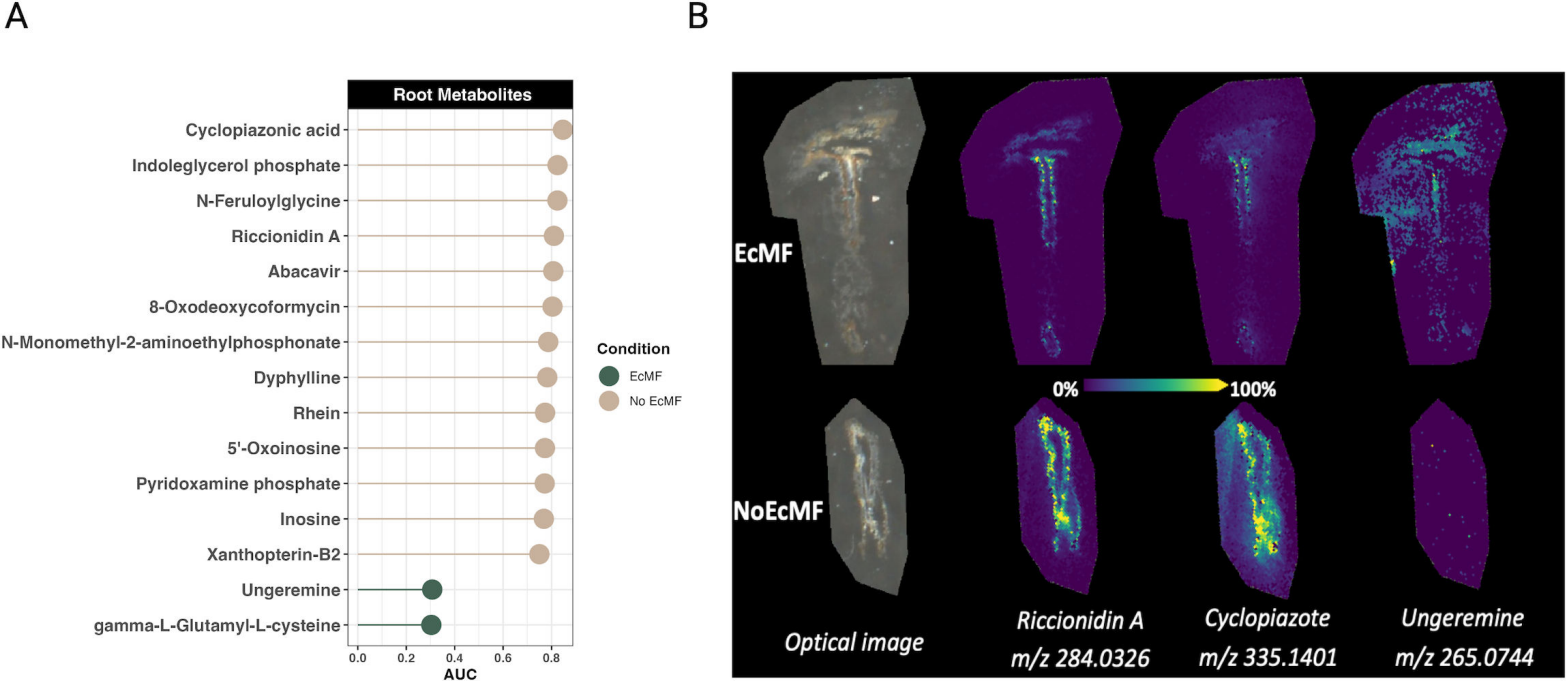
MALDI-FTICR detected root metabolites. Metabolites that were significantly enriched in plant roots that either were colonized by EcMF (green) or were not colonized by EcMF (tan) are shown. (**A**) Area under the curve values represent how enriched metabolites were between conditions (i.e., EcMF versus No EcMF). The ions detected for all the metabolites listed represent a single metabolite according to the KEGG database. (**B**) MALDI ion root images comparing the localization and intensity of selected metabolites between treatments. Note that all annotations are confirmed only by MS1 at the exact molecular formula level. For a comprehensive list of all the ions and their potential metabolite annotations, see Tables S4 and S5.

While our previous measurements characterized chemical changes in soils beyond the rhizosphere, we also used mass spectrometry imaging to understand how the presence of EcMF changes the chemical environment within roots and at the root-soil interface. Using this approach, we compared root metabolites between experimental conditions (i.e., EcMF or No EcMF) with MALDI-FTICR, using the area under the curve from receiver operating characteristic analyses for finding discriminant ions between two treatments and METASPACE for tentative molecular annotation of ions. Since MALDI-FTICR measures accurate mass ions (i.e., their *m*/*z*), and distinct metabolites can have identical masses (i.e., mass isomers), we report potential metabolites that were enriched and depleted between conditions. A total of 330 ions (representing 1,022 potential metabolites) were annotated by METASPACE in our analysis (Table S4), and 85% were not significantly enriched or depleted between conditions. Only two ions, representing two metabolites annotated in the KEGG database, were enriched in the presence of EcMF: gamma-L-glutamyl-L-cysteine (*m*/*z* 249.0550) and ungeremine (*m*/*z* 265.0744). In contrast, 49 ions (representing 294 potential metabolites) were enriched in the absence of EcMF (Table S5). Of these 49 ions, 13 of them correspond to a single metabolite by the KEGG database (Fig. 6A). In addition, we captured images of both non-ectomycorrhizal and ectomycorrhizal roots to observe the spatial distribution of metabolites. We observed clear differences in both the intensity and localization of several differentially enriched metabolites (Fig. 6B). The results from this analysis suggest that EcMF modify the energy dynamics of the host plant (i.e., reducing the production of primary and secondary metabolites). However, given the limited sample size, we were able to detect only a few statistically enriched metabolites in the root compartment.

## DISCUSSION

Many investigations have examined plant-microbe interactions that occur solely at the root-soil interface (i.e., the rhizosphere or rhizoplane), yet whether ectomycorrhizal fungi (EcMF) restructure soil food webs and bacterial communities has remained an outstanding question that lacked experimental evidence (59). This is an important point of inquiry when we consider that (i) the interplay between soil metabolites and soil microbiota largely determines the fate of plant communities (60, 61); (ii) EcMF may functionally extend the rhizosphere compartment and impact soil food webs (23); and (iii) as recipients of approximately 13% of plant primary productivity (62), EcMF are major conduits and transformers of C inputs to soils. To address this knowledge gap, we directly tested how the presence of EcMF changes soil bacterial communities and substrate availability using a multi-omics approach. Our analyses show that the presence of EcMF has significant effects on bacterial diversity and richness, bacterial taxa-function relationships, and biochemistry in soils and at the root-soil interface. We, therefore, have begun elucidating the potential for EcMF to modify soil food web structure and offer critical insights into one of the most widespread mutualisms in the world.

### The effects of EcMF on bacterial richness and beta diversity

Many interactions among microbes in complex systems have been inferred using correlational analyses of co-occurrence data from DNA sequencing. Here, we were able to use an experimental approach to reveal the direct responses of bacteria to soils amended with EcMF. Because these seedling microcosms were grown in a sterilized pure sand substrate, this approach allowed us to disentangle confounding differences in soil chemistry or litter quality that may have driven bacterial associations with EcMF trees documented in field studies (6, 13). This finding indicates that the presence of EcMF hyphae in soils has direct effects on the composition and structure of bacterial communities. In soils amended with EcMF, bacterial richness was increased, and community composition changed—both in terms of taxonomic composition and increased homogeneity. Others have found that soils inhabited by either AMF or saprotrophic fungi tend to have higher microbial richness than soils without these fungi (31, 63, 64). The fact that we see similar trends for soils amended with EcMF (Fig. 1A) supports the notion that diverse fungal types—not only EcMF—may relax bacterial competition or create new niches. This contrasts somewhat with the strongly antagonistic relationship inferred between bacteria and fungi in a recent global metagenomic survey (65). In terms of beta diversity, we also observed significant differences between experimental conditions. Soils amended with EcMF were taxonomically more homogenous than those without EcMF (Fig. 1B; Fig. S1 and S2). This observation contrasts with findings derived from AM fungus systems (32). However, it aligns with previous evidence showing that EcMF tend to cultivate more homogenous microbial communities (66). Why this happens has yet to be addressed experimentally, but EcMF and AM fungi may foster the development of different bacterial soil communities due to their differences in fungal growth, physiology, and plant root colonization dynamics (1). One possible hypothesis is that a high degree of underground connectivity generated by EcMF mycelium may create a spatially homogenous environment that leads to more predictable assemblies of bacterial communities. While knowing the specific nature of these interactions will require additional experiments, our data collectively point toward a complex suite of interactions between bacteria and EcMF that appear to transcend simple competition.

### Linking greenhouse and field studies: similar patterns of bacterial-EcMF partnerships

Linking laboratory and field observations has been a challenge in microbial ecology (67). However, the bacterial taxa that we identified in soils amended with EcMF align largely with those that previous studies have identified in natural *Pinus muricata* forests (18, 68). At the phylum level, Proteobacteria and Bacteroidota had a higher relative abundance in soils amended with EcMF compared to those without EcMF (Fig. S2). Many genera in these phyla were also significantly enriched in EcMF-amended soils (Fig. 1C). For instance, amplicon sequence variants within the genera *Bradyrhizobium*, *Conexibacter*, and *Reyranella* were enriched in EcMF-amended soils in this experiment and were shown recently to function as strong, positive predictors of EcMF relative abundance along a climate-latitude gradient (18). Importantly, the genus *Bradyrhizobium* was shown to correlate positively with plant biomass and the colonization of EcMF on plant roots (18), reinforcing the notion that EcMF-amended soils generate soil conditions that are advantageous to bacterial taxa that in turn benefit EcMF growth dynamics. Likewise, the preserved field-to-lab connections that we observed between our current study and those conducted previously (18, 68) demonstrate that some relationships between EcMF and bacteria can be recapitulated independently of soil characteristics and microbial diversity. Yet, particular bacterial genera—and species within these genera—likely have higher intra-genus and intra-species variability in their lifestyle strategies, which reinforces the notion of varying strain-specific interaction strengths between and among plants and microbes (69, 70). Overall, these observations suggest that EcMF play a key role in structuring bacterial communities via direct fungal and tripartite symbioses.

### Estimates of how EcMF shape the functional potential of soil bacterial communities

In line with our observation that EcMF drive changes in the taxonomic composition of bacterial taxa (Fig. 1; Fig. S1 and S2), we also observed changes to the inferred functional potential of these bacterial taxa (Fig. 2; Fig. S3). Of note, soils amended with EcMF nearly doubled the relative abundance of bacterial taxa likely to harbor the gene needed to reduce plant ethylene levels (i.e., 1-aminocyclopropane-1-carboxylate, ACCd). This well-known gene has been documented extensively as one of the primary plant growth-promoting features of many beneficial bacteria (71). The fact that we observe an increased relative abundance of this gene in the presence of EcMF (Fig. 2) could indicate a role in tripartite symbioses. While the exact mechanism is not immediately clear, plant ethylene has been shown to alter EcMF colonization—often leading to a partial reinforcement of plant cell walls that could restrict mycorrhizal symbioses (72). By selecting for ACCd-producing bacterial taxa, EcMF could therefore potentially increase their ability to colonize plant roots. Moreover, this potential fine-tuning event may also serve to regulate other plant hormones (e.g., auxin), particularly during the early stages of symbiosis (73). Alternatively, the role of bacterial ACCd may be context-dependent—as others have shown that harboring this metabolic feature does not always lead to plant growth benefits (70). Therefore, additional work is required to determine whether ACCd shapes tripartite symbioses or is indicative of commensalistic bacterial-fungal interactions.

Aside from changes to the relative abundance of ACCd in bacterial soil communities, we also observed differences that may be linked to nutrient consumption and bacterial colonization-lifestyle patterns. For example, the operon involved in the degradation of phenylacetic acid (*paa*A, *paa*B, *paa*C, *paa*I, *paa*X, and *paa*Y) was enriched in our EcMF condition based on the PICRUSt2 analysis (Fig. 2). Others have noted that phenylacetic acid is a common metabolite released at fungal surfaces, suggesting that EcMF may select for bacteria that can actively degrade and use this compound for energy production (74, 75). Likewise, we see clear indications of changes to nitrogen (N) cycling potential. The gene coding for asparagine synthase (glutamine-hydrolyzing) is essential for plant N assimilation, and this gene is enriched in bacteria in soils without EcMF amendments (Fig. 2). This paired with the enrichment of bacterial taxa (in soils without EcMF amendments) that play key roles in nitrification (i.e., *Nitrobacter* and *Nitrosospira*) in non-mycorrhizal soils suggest that the way EcMF can change the local N economy, independent of the soil environment. In addition to modifying N economics, the presence of EcMF selected for bacterial communities with a higher relative abundance of genes to biosynthesize trehalose (Fig. S3B)—a carbohydrate known to play osmo-protective, thermal-protective, and energy-generating roles (76). Other mechanisms of bacterial colonization-lifestyle strategies may also be driven by EcMF. For instance, soils without EcMF amendments drove the proliferation of bacteria with a higher relative abundance of pilT genes (involved in movement along surfaces), and this observation aligns with the increased motility potential of these bacterial communities (Fig. 1E). These data therefore suggest that EcMF may function as spatial carriers for bacterial cells and that they select for bacteria that are strong hyphal colonizers yet not strong dispersers, which is likely a product of the highly interconnected belowground environment that EcMF can foster. Taken together, our estimates of bacterial functions provide evidence that (i) EcMF may restructure carbon and N soil economies, and (ii) EcMF colonization may select for specific bacterial motility strategies. However, additional functional profiling analyses (e.g., meta-transcriptomics) are required to fully understand how EcMF change the community-wide functions of soil bacteria.

### Non-lipid soil metabolite profiles and their potential links to bacterial community functions

When we examined the metabolic profile of bulk soils, we found that the presence of EcMF drove the enrichment of 17 soil metabolites, and three of these (D-sorbose, D-ribose, and scyllo-inositol) were consistently and exclusively present in soils amended with EcMF (Fig. 3; Fig. S4). Links between these enriched metabolites and the metabolic potentials of bacterial communities were sparse, but several connections were evident. For example, several of them were either precursors (i.e., glutamic acid), intermediates (i.e., fumaric acid), or inhibitors (i.e., malonic acid) in the tricarboxylic acid (TCA) cycle, and the community metabolic potentials for EcMF soil bacteria were predicted to contribute substantially to the metabolism of the same (i.e., fumaric acid) or complementary (e.g., succinic acid) metabolites in the TCA cycle. EcMF-enriched bacterial taxa (e.g., *Bradyrhizobium*, *Rhizobium*, and *Shinella*) were also identified as potentially major contributors to these metabolic processes (Fig. 1C and 5; Table S3), suggesting that (i) EcMF alter the biochemical flux of energetic metabolites available to bacterial communities in bulk soils, and (ii) EcMF-specialist bacterial taxa that have been identified in field studies (18, 68) are primary players in these processes. Others have noted how EcMF modify the production of TCA cycle metabolites in plant host roots (77–79), but here we show that these modifications extend into the bulk soils and may drive both bacterial coexistence and taxonomic dominance. In addition, L-threonine was highly enriched in soils amended with EcMF, and CMP estimates suggest that several key bacterial taxa drive its metabolism (Fig. 5; Table S3). The functional role(s) of L-threonine in these contexts suggest greater bacterial protein synthesis and biofilm formation—both of which modify bacterial physiology and growth dynamics (80). Therefore, the primary takeaways from these soil metabolite data are that EcMF facilitate the production of key carbon sources and subsequently influence the community assembly of soil bacteria—many of which have been independently confirmed to mediate tripartite symbioses in both field and lab studies (18, 68).

### Changes to soil lipids: building blocks, antimicrobials, and signaling molecules

Our soil lipid data also indicate that EcMF significantly change the community-wide lipid profiles of bulk soils and drive the differential enrichment of 37 lipids (Fig. 4), which collectively may have cascading effects on soil food webs and the composition of bacterial communities. Several differentially enriched lipid subgroups (i.e., diacyl glycerolipids with 16:0/18:2 or 16:1/18:2 tails and glycerophospholipids with 18:2/18:2 tails) have been identified previously as signatures of mycorrhization, fungal colonization, and fungal biomass (81, 82). Therefore, it is not surprising that we see these lipid species enriched in soils amended with EcMF. But how do these changes to soil lipid profiles shape bacterial communities? Many of these lipids serve structural roles within fungal cell walls and membranes (i.e., glycerolipids and glycerophospholipids), and some of them that are frequently found in fungal membranes have been suggested to have antibiotic properties (i.e., sphingolipids) or trigger the production of antimicrobial peptides (83). Therefore, the three hypotheses explaining these differences are that (i) these lipids provide structural and energetic materials for bacterial cells (i.e., trophic transfer, perhaps from necromass); (ii) some of them also function as bactericidal or bacteriostatic compounds; and (iii) they may mediate cellular communication between and among plants and microbes. For instance, lipids represent a large fraction of carbon pools in both the rhizosphere and bulk soils (83), and trophic transfer events and cell-cell communication are presumably common in both soils (84–86). In addition, the antimicrobial properties of sphingolipids can limit the formation of biofilms (83), and each of the differentially abundant sphingolipids that we observed in our analysis was enriched in soils without EcMF (Fig. 4). This paired with the enrichment of L-threonine—a promoter of bacterial biofilm formation—in EcMF-amended soils and estimates that bacterial taxa primed to metabolize L-threonine are enriched in EcMF-amended soils suggest that EcMF may facilitate the production of biofilms. Together, these data provide mechanistic insights into how the presence of EcMF can restructure bulk soil carbon economics, modify soil food webs, and provide a selective force that drives the establishment of bacterial communities.

### EcMF-driven root metabolite production: evidence for pathogen suppression and energetic modifications

Nutrient availability and composition tend to differ drastically between plant root and soil compartments (87). When we profiled the metabolites of both colonized and uncolonized Bishop pine roots (Fig. 6), we observed significant differences between the metabolites that were enriched—and these metabolites were distinct from those that we observed in soils (independent of treatment type; see Fig. 3 and 4). The most prominent observation was that uncolonized roots had many more metabolites that were enriched compared to EcMF-colonized roots. The function(s) of these metabolites are varied, but common themes are evident. For example, many of them (N-feruloylglycine, abacavir, 8-oxodeoxycoformycin, N-monomethyl-2-aminoethylphosphonate, dyphylline, oxoinosine, and xanthopterin-B2) have unknown functions in the context of plant biology or microbial ecology. Yet, several of them (abacavir, 8-oxodeoxycoformycin, and dyphylline) have reported anti-viral (e.g., HIV), anti-cancer, or bronchodilator functions (88–91). Other detected metabolites, however, yield clearer interpretations in the context of our system. Cyclopizaonic acid, for instance, is a well-known mycotoxin that is produced by *Penicillium cyclopium* (92). The fact that this metabolite is enriched in the non-mycorrhizal roots suggests that the presence of EcMF suppresses the colonization and proliferation of *Penicillium* sp. or—more conservatively—the accumulation of cyclopiazonic acid (Fig. 6B). Similarly, riccionidin A is a known anthocyanidin and photo-protectant that plays a critical role in cell wall-associated defense (93). The enrichment of this metabolite in the absence of EcMF suggests that EcMF may dampen plant host defenses or reduce the presence of pathogens and reconfigure energy dynamics in and around the plant root cell walls. Several other lines of evidence suggest further that EcMF alter the flux of energetic pathways within plant roots, such as the enrichment of inosine (alters RNA structure and can lead to mistranslation), indoleglycerol phosphate (branch point in the biosynthesis of the plant growth hormone indole-3-acetic acid), and pyridoxamine phosphate (prerequisite to amino acid synthesis) in non-mycorrhizal roots (94–96). In contrast to the variety of metabolites enriched in the roots of plants grown in the absence of EcMF, the roots of plants grown in the presence of EcMF had only two enriched metabolites (i.e., ungeremine and gamma-L-glutamyl-L-cysteine). Functionally, ungeremine has been described as an anti-mold biofungicide, whereas gamma-L-glutamyl-L-cysteine is a key intermediate in the glutathione synthesis pathway (97). Therefore, these results suggest that the presence of EcMF may limit the accumulation of mold (i.e., pathogen suppression) and drive the synthesis of strong reactive oxygen species scavenging molecules, respectively (98, 99). Taken together, our root metabolite data illustrate that the presence of EcMF may both modify plant host energy dynamics and suppress the outgrowth of non-mycorrhizal fungi.

Though many of the observations in our present study align with those from previous field studies (18, 68), several considerations should be undertaken moving forward. For instance, bridges between bacterial community assembly, genetic regulation, and metabolite production require additional experimental work to determine the causal factors that support bacterial-fungal interactions and connect multi-omic data sets. Functional genetics approaches, for example, that leverage both bacterial and fungal mutant libraries to test the effect of the presumptive functions we have revealed in this study would enhance our understanding of the genetic and metabolic determinants of bacterial-fungal-plant interactions. Likewise, efforts to harmonize multi-omic data sets would clarify the relationships among bacterial gene functions, soil metabolites, root metabolites, and soil food webs in general (100). Our data sets here do reveal how the presence of EcMF can alter resource availability and modify bacterial communities in ways that suggest an overall reshaping of soil food webs. However, our data sets cannot reveal the microbial taxa that are producing and consuming specific metabolites. Methods to do so still require substantial developments for the complex communities found in plant microbiomes. Finally, our observations are limited to a specific time point of plant development. Though previous field observations (18, 68) across different climates, latitudes, and forest maturity confirm many of the bacterial taxonomic trends that we observed here, efforts to understand how soil food webs change in the presence of EcMF across plant developmental time will help resolve the complex mechanisms that likely shape these community-level interactions.

### Conclusion

How EcMF drive changes in soil biogeochemistry has received little experimental attention. Yet, emerging evidence suggests that soil chemical structure is the by-product of bacterial and fungal biogeochemistry (101, 102). Using a multi-omic approach, we show here that the presence of a keystone guild (i.e., EcMF) can impact soil lipid and non-lipid metabolites, soil food webs, and the bacterial communities that develop. Our work, therefore, demonstrates how the presence of EcMF can modify plant host roots and have substantial consequences on the chemical and bacterial community composition of bulk soils. In broader terms, our work adds to the emerging set of fundamental differences between arbuscular mycorrhizal and EcMF forests (2), which further emphasizes the need to account explicitly for the functional differences of these forests and the theory and models that surround them.

## Data Availability

All data and code generated in the current study can be found in either NCBI GenBank SRA (BioProject accession number PRJNA1100605), https://github.com/LouisBerrios, the main text, or the supplemental material.

## SUPPLEMENTAL MATERIAL

Supplemental figures msystems.00369-24-s0001.docx Figures S1 to S6.

Supplemental tables msystems.00369-24-s0002.xlsx Tables S1 to S5.

ASM does not own the copyrights to Supplemental Material that may be linked to, or accessed through, an article. The authors have granted ASM a non-exclusive, world-wide license to publish the Supplemental Material files. Please contact the corresponding author directly for reuse.
