# Supplementary material for: Ectomycorrhizal fungi alter soil food webs and the functional potential of bacterial communities
Source: mSystems. 2024 May 8;9(6):e00369-24. doi: 10.1128/msystems.00369-24 (PMC11237468; doi:10.1128/msystems.00369-24)
Supplement: Supplemental figures — Figures S1 to S6. [file msystems.00369-24-s0001.docx]

**Supplementary Figures**

**Ectomycorrhizal fungi alter soil food webs and the functional potential of bacterial communities**

Louis Berrios^✉1^, Glade D. Bogar^2^, Laura M. Bogar^3^, Andressa M. Venturini^1^, Claire E. Willing^1,4^, Anastacia Del Rio^1^, T. Bertie Ansell^1,5^, Kevin Zemaitis^6^, Marija Velickovic^6^, Dusan Velickovic^6^, Peter T. Pellitier^1^, Jay Yeam^1^, Chelsea Hutchinson^6^, Kent Bloodsworth^6^, Mary S. Lipton^6^, Kabir G. Peay^1,7^

^1^ Department of Biology, Stanford University, Stanford CA, 94305, USA

^2^ Kellogg Biological Station, Michigan State University, 3700 E Gull Lake Dr, Hickory Corners MI 49060, USA

^3^ Department of Plant Biology, University of California, Davis, 605 Hutchison Dr., Davis CA, 95616, USA

^4^ School of Environmental and Forest Sciences, University of Washington, Seattle WA, 98195, USA

^5^ Division of CryoEM and Bioimaging, SSRL, SLAC National Accelerator Laboratory, Menlo

Park CA, USA

^6^ Earth and Biological Sciences Directorate, Pacific Northwest National Laboratory, Richland WA, 99354, USA

^7^ Department of Earth System Science, Stanford University, Stanford CA, 94305, USA

✉ Lead Contact: Louis Berrios; Email: [berriosl@stanford.edu](mailto:berriosl@stanford.edu)


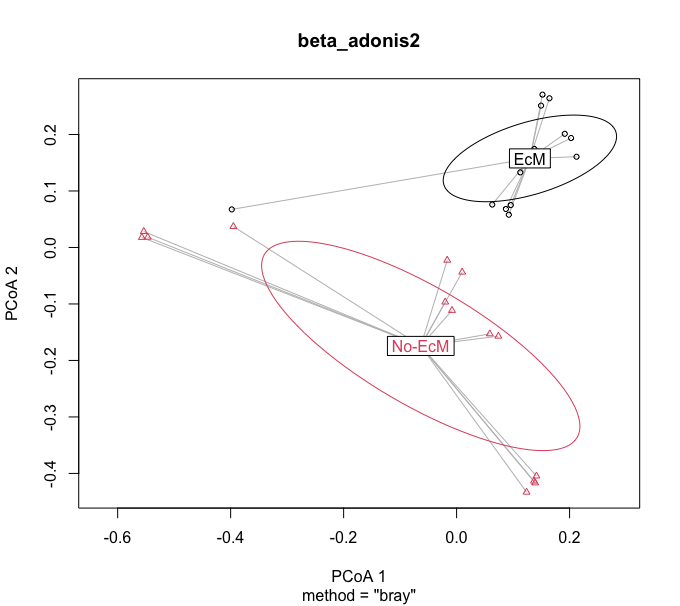


**Figure S1. Principal component analysis comparing soil bacterial communities either in the presence of ectomycorrhizal fungi (EcM) or in the absence of EcM fungi (No-EcM).** Elliptical hulls represent 95% confidence intervals. The betadispr function in the vegan package was used to conduct this analysis.


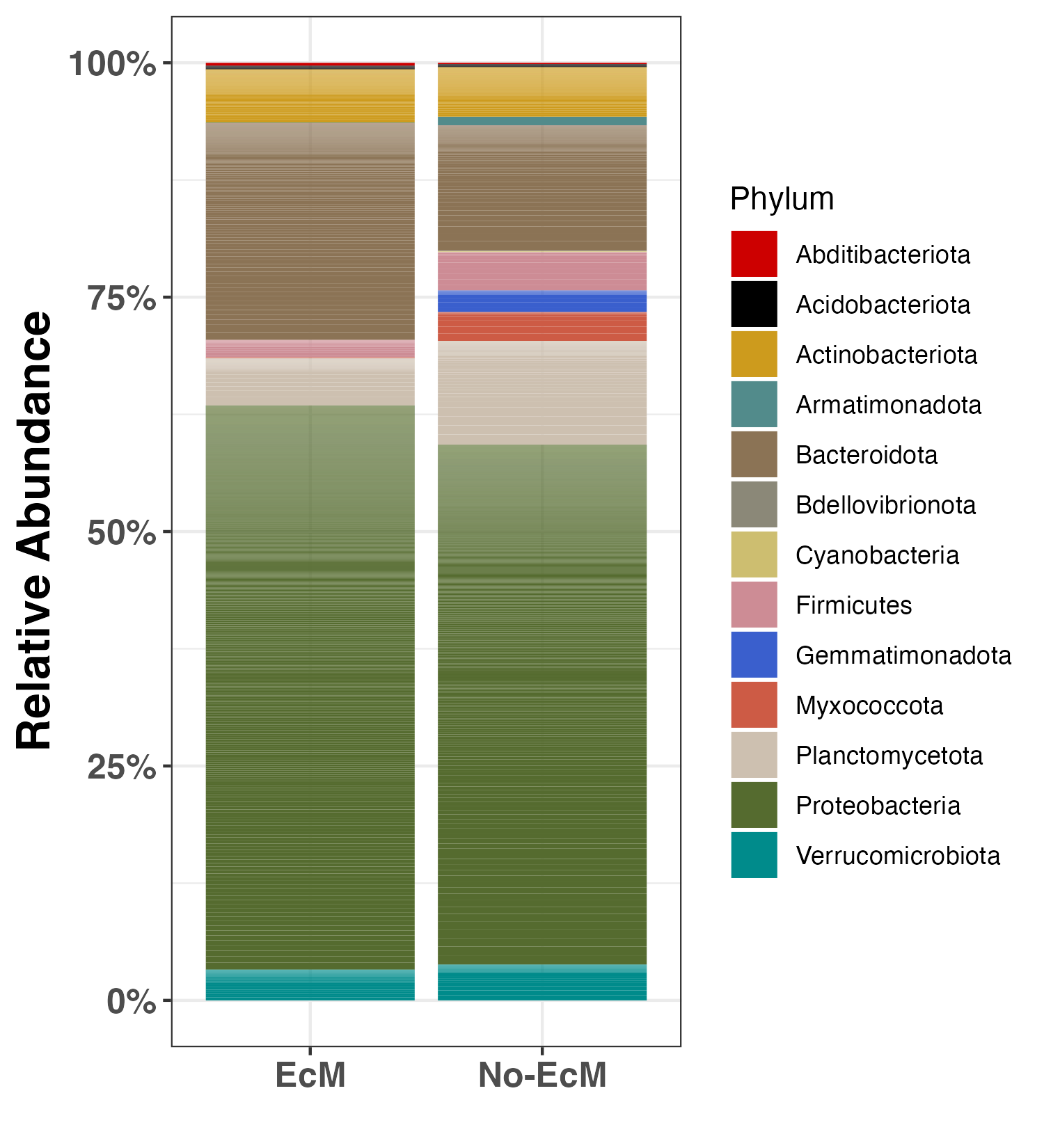


**Figure S2. Relative abundance bar plot comparison between bacterial communities either in association with ectomycorrhizal fungi (EcM) or without EcM (No EcM).** Bacterial taxa are grouped and colored by their respective phylum designation.


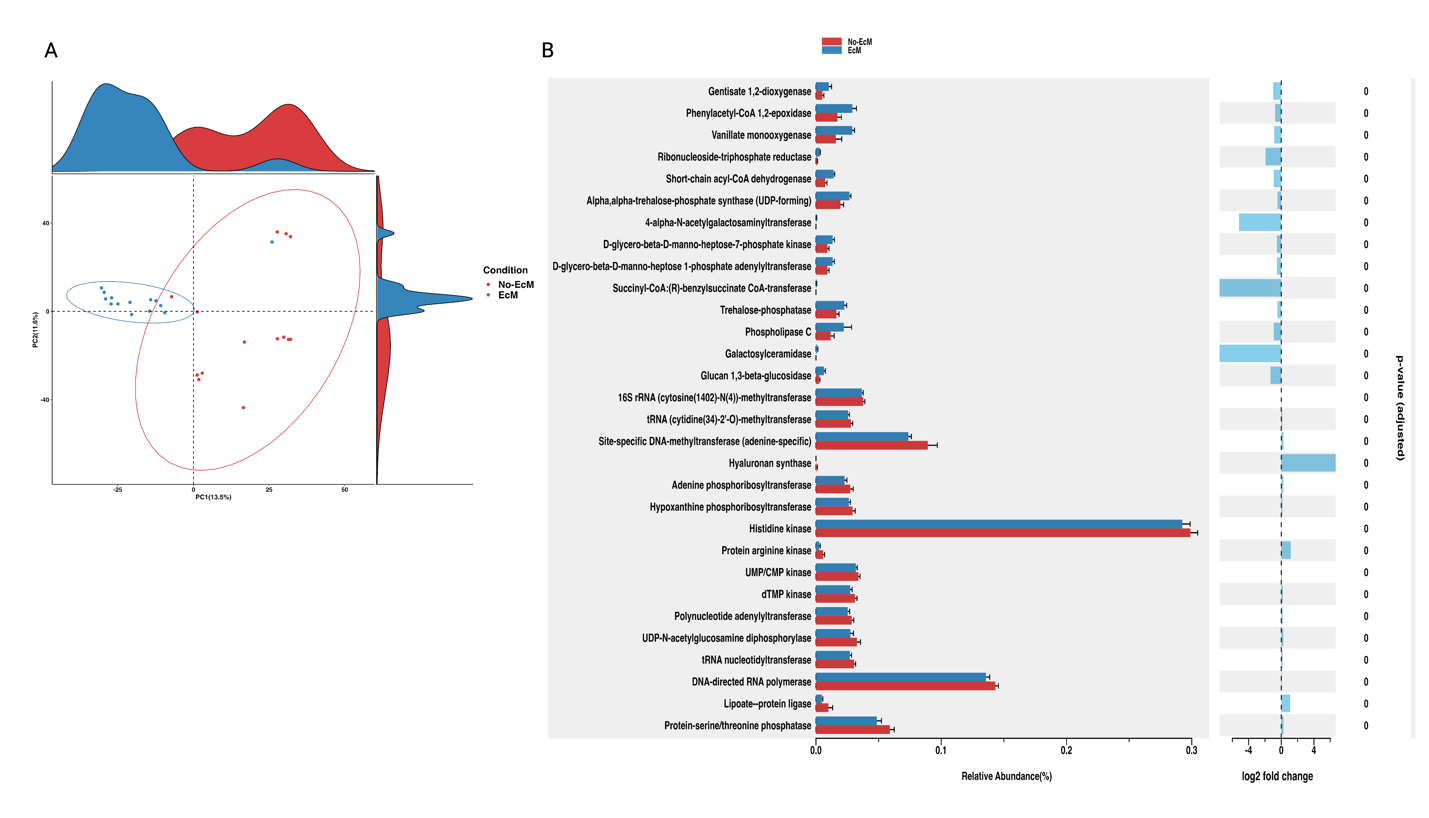


**Figure S3.** **PICRUSt2 analysis of bacterial communities in the mycorrhizosphere (with EcMF) compared to the rhizosphere (without EcMF). A)** Principal component analysis demonstrating the functional differences (EC numbers) between bacterial communities based on treatment. Ellipses indicate confidence intervals of 95%. **B)** The top 30 EC terms that were differentially abundant between treatments according to DESeq2 (p < 0.05; p-adjusted using Holm-Bonferroni method). Errors bars indicate confidence intervals of 95%. Adjusted p-values and the relative log_2_ fold-change are provided adjacent to the relative abundance of the top 30 gene products, and EC numbers are listed alongside their corresponding gene product name. See Table S1-S2 for a comprehensive list of the KO features and EC numbers detected in our analyses.


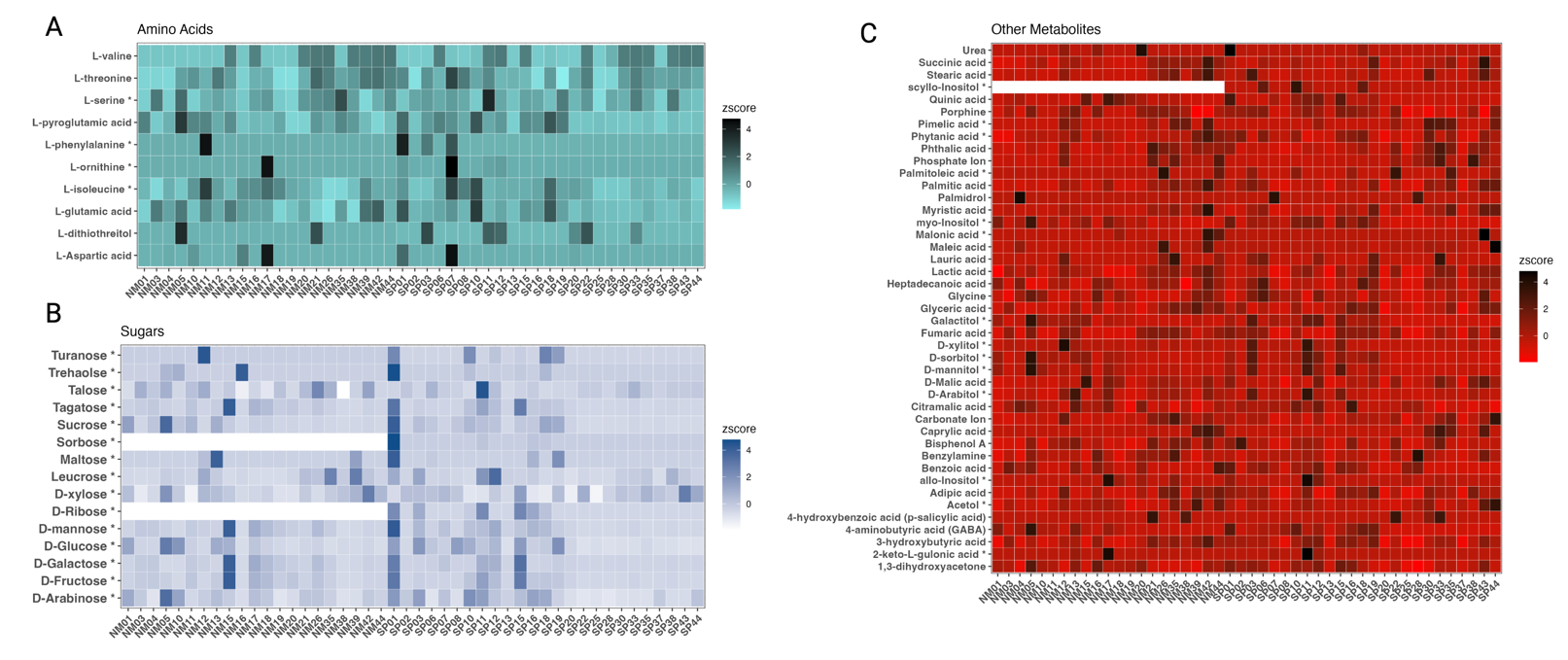


**Figure S4. Heatmaps of soil metabolites from samples with either ectomycorrhizal fungi (SP samples) or samples without ectomycorrhizal fungi (NM samples). A)** Amino acids **(B)** sugars, and **(C)** other metabolites. Z scores are shown for each heatmap. Tiles colored solid white indicate a complete absence of the corresponding metabolite.


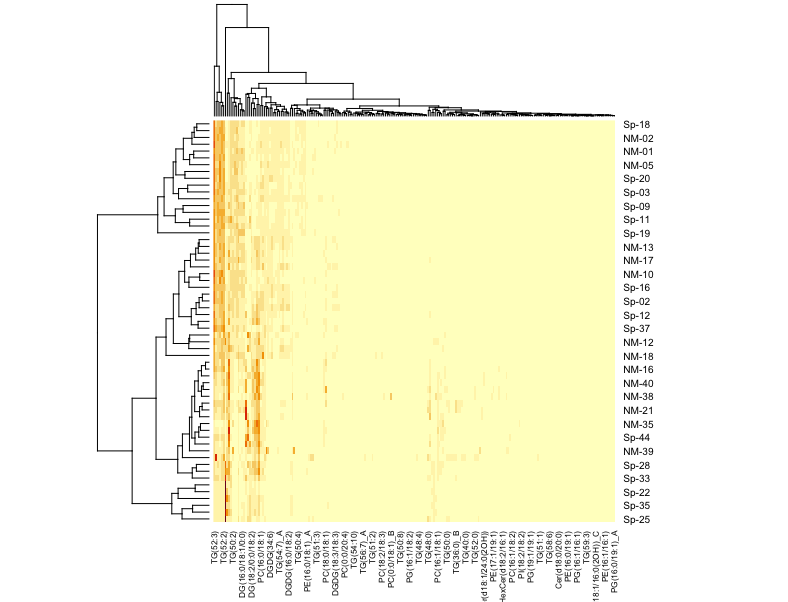


**Figure S5. Heatmap of lipidomics dataset.** Lables with a ‘SP’ are samples with ectomycorrhizal fungi. Labels with a ‘NM’ are samples without ectomycorrhizal fungi. Lipids are grouped based on the structural similarity. The lipid species designations are as follows: Cer = Ceramide; HexCer = hexosylceramide; PC = phosphatidycholine; PC-Lyso = lyso-phosphatidycholine; PE = Phosphatidylethanolamine; PG = phosphatidylglycerol; PI = phosphatidylinositol; DGDG = digalactosyldiacylglycerol; DGTSA = diacylglyceryltrimethylhomo-serine; SQDG = sulffoquinovosyl diacylglycerol; DG = diacylglycerol; TG = triacylglycerol. The lipid class designations are as follows: Cer and HexCer = Sphingolipids; PC, PC-Lyso, PE, PG, and PI = Glycerophospholipid; DGDG, DGTSA, SQDG, DG, and TG = Glycerolipid.


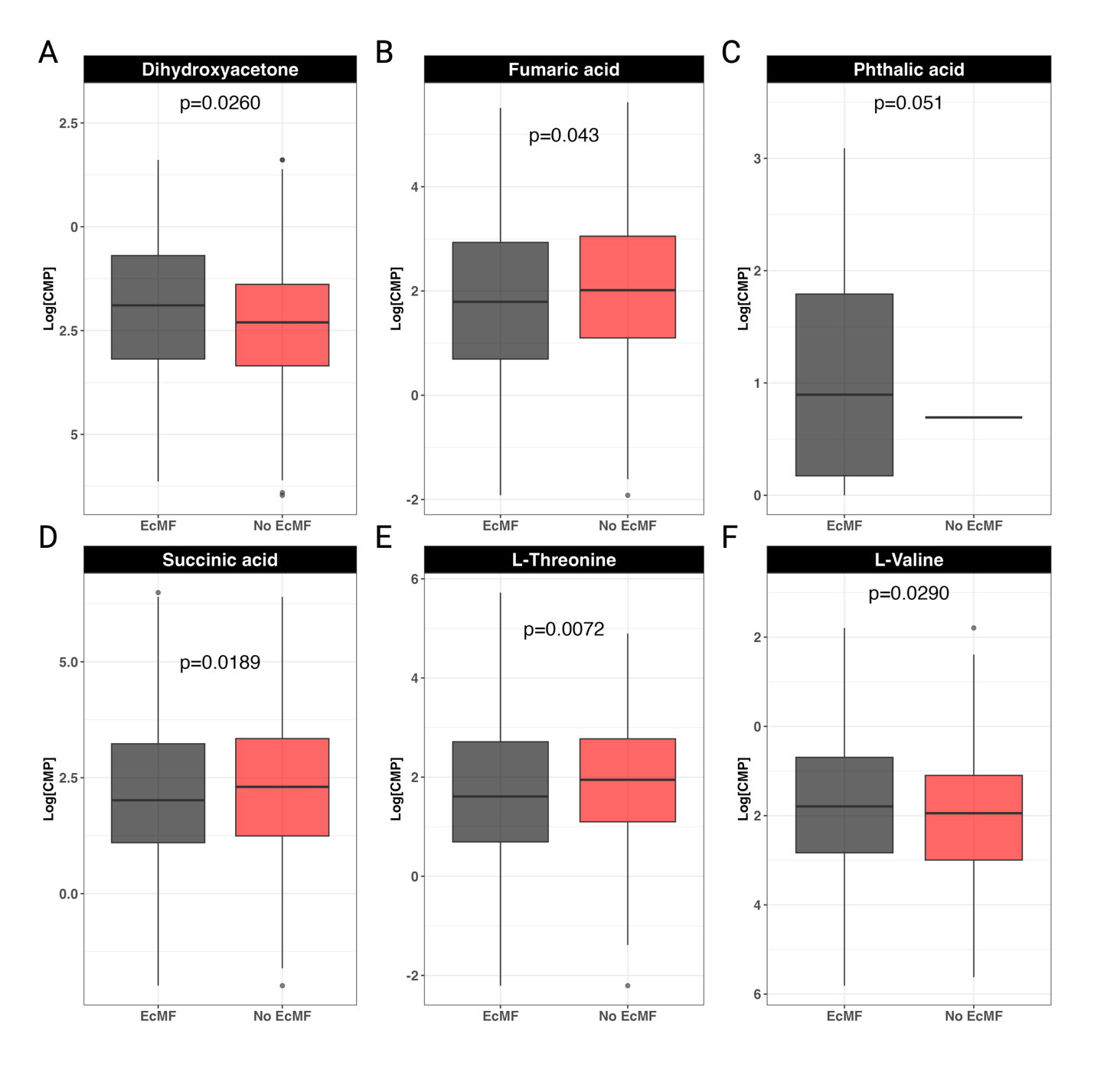


**Figure. S6. MIMOSA2 community metabolic potential (CMP) comparisons.** All CMPs were aggregated based on experimental condition (i.e., soils inoculated with ectomycorrhizal fungi [EcMF] and those not inoculated with EcMF [No EcMF]). Welch’s t tests were conducted to compare CMPs between conditions. P-values are shown for each comparisons.
